# ID 241 - Desafios e Perspectivas para Definir um Limiar de Custo-Efetividade na Saúde Suplementar no Brasil

**DOI:** 10.5327/2237-9622.2025.v34s1.241

**Published:** 2025-11-25

**Authors:** Silvana Marcia Bruschi Kelles, Marcus Carvalho Borin, Mariana Michel Barbosa, Carina Rejane Martins, Daniel Pitchon dos Reis, Geraldo José Coelho Ribeiro, Júlia Teixeira Tupinambás, Karina de Castro Zocrato, Lélia Maria de Almeida Carvalho, Marcela Pinto de Freitas, Maria da Glória Cruvinel Horta, Mariza Cristina Torres Talim, Sergio Adriano Loureiro Bersan

**Keywords:** limiar de custo-efetividade, ICER, QALY, saúde suplementar

## Abstract

**Introdução:**

No Brasil, o Sistema Único de Saúde (SUS) atende a maior parte da população, mas cerca de 25% dos brasileiros têm contratada também a assistência pela saúde suplementar, regulada pela Agência Nacional de Saúde Suplementar (ANS). Diferentemente da Comissão Nacional de Incorporação de Tecnologias no SUS (Conitec), que estabeleceu o limiar de R$ 40 mil, equivalente a um PIB per capita, por ano de vida ajustado pela qualidade (QALY) para a incorporação de tecnologias, a ANS não possui diretrizes claras para a avaliação de custo-efetividade. Essa ausência cria uma lacuna significativa no processo de incorporação de medicamentos no setor privado, levando a variações no acesso e risco à sustentabilidade financeira do sistema suplementar. A criação de um limiar de custo-efetividade adaptado à saúde suplementar poderia aumentar a eficiência do sistema e garantir maior equidade no acesso a tratamentos de alto custo. Este estudo avalia a razão de custo-efetividade incremental (ICER – do inglês, ) por QALY de medicamentos incorporados pela saúde suplementar, após discussão no âmbito da ANS, e compara esses valores ao limiar da Conitec, destacando as implicações para sustentabilidade do setor e acesso dos beneficiários.

**Método:**

Foram analisados dados de reembolso de medicamentos submetidos à ANS, com foco na ICER por QALY de diferentes tratamentos. As ICERs foram calculadas com base nos custos dos tratamentos e nos benefícios em termos de QALYs. Medicamentos como talazoparibe, enzalutamida, lenvatinibe e gilteritinibe, utilizados em tratamentos oncológicos, foram incluídos na análise. Os resultados foram comparados ao limiar de R$ 40 mil por QALY estabelecido pela Conitec e a limiares internacionais, a fim de identificar medicamentos cujas ICERs excedem os valores de referência.

**Resultados:**

Os resultados revelaram uma discrepância significativa entre as ICERs observadas no setor privado e o limiar estabelecido pela Conitec. Por exemplo, o talazoparibe, para o câncer de mama avançado, apresentou uma ICER de R$ 619.900 por QALY, enquanto a enzalutamida, para o câncer de próstata metastático, atingiu R$ 366.490 por QALY. Medicamentos como o gilteritinibe, para leucemia mieloide aguda, apresentaram ICER de R$ 492.250 por QALY. Em contraste, intervenções como a diálise peritoneal automatizada mostraram ICERs bem abaixo do limiar da Conitec, com R$ 6.850 por QALY.

**Conclusão:**

A variabilidade na magnitude da ICER/QALY evidencia a falta de critérios econômicos para o setor suplementar. A ausência de um limiar de custo-efetividade cria incertezas na incorporação de medicamentos de alto custo, potencialmente sobrecarregando financeiramente as contraprestações dos beneficiários. O estabelecimento de um limiar formal para o setor suplementar, alinhado às práticas internacionais e às diretrizes da Conitec, pode otimizar a alocação de recursos e aumentar o acesso a esse sistema.

